# Can Diet Help Non-Obese Individuals with Non-Alcoholic Fatty Liver Disease (NAFLD)?

**DOI:** 10.3390/jcm6090088

**Published:** 2017-09-19

**Authors:** Hamid A. Merchant

**Affiliations:** Department of Pharmacy, School of Applied Sciences, University of Huddersfield, Queensgate, Huddersfield HD1 3DH, UK; hamid.merchant@hud.ac.uk; Tel.: +44-(0)-1484-472387; Fax: +44-(0)-1484-472182

**Keywords:** diabetes risk, insulin resistance, fatty liver disease, hyperinsulinemia, NAFLD, NASH, intensive lifestyle change program

## Abstract

Subjects diagnosed with non-alcoholic fatty liver disease (NAFLD) or hepatic steatosis are usually obese or overweight. NAFLD has also been reported in many non-obese healthy subjects as an incidental finding during imaging. Subjects with early-stage NAFLD who are otherwise healthy are often left unmanaged in current clinical practice; it is not clear if an early intervention in those individuals would be of any benefit in preventing NAFLD progression to more serious conditions. Since many of these subjects are non-alcoholic and have a normal body mass index (BMI), an intensive lifestyle change program is not usually recommended. This report presents an otherwise healthy non-alcoholic subject with incidental NAFLD having a normal BMI and a waist circumference below 90 cm who successfully reversed his condition by undertaking a lifestyle intervention. The case report is expected to encourage large cohort studies to substantiate the benefits of dietary interventions in alleviating hepatic steatosis among non-obese individuals.

## 1. Introduction

Non-alcoholic fatty liver disease (NAFLD) or hepatic steatosis is usually common in subjects who are obese or overweight. It is very common in non-obese South Asians with considerably lower body mass index with manifestations of abdominal adiposity. Recently, these subjects are often categorized as “lean NAFLD” [1] or “normal weight metabolically obese” [2]. NAFLD is very common and estimates show that one in three individuals in UK may have some fatty deposits in the liver [3]. These abnormal deposits of lipids within hepatic cells hampers homeostasis of triglyceride synthesis and storage. NAFLD has been shown to be a major risk factor for diabetes and cardiovascular disease; the soaring numbers of diabetes and cardiovascular events is already a major burden on national health services. NAFLD may cause liver damage if left uncontrolled and may progress into steatohepatitis (NASH) and even cirrhosis. The research has shown that dietary interventions have a close relationship with metabolic diseases and may also benefit these patients. This report presents a case of an otherwise healthy non-obese subject, recently diagnosed with an early stage NAFLD who undertook a low-carb diet and was successful in reversing his condition. This is in support to the existing evidence in scientific literature that dietary modulations can be very beneficial in treating NAFLD and reducing the burden in hepatology, diabetes and cardiology. 

## 2. Case Report 

**Figure 1:**
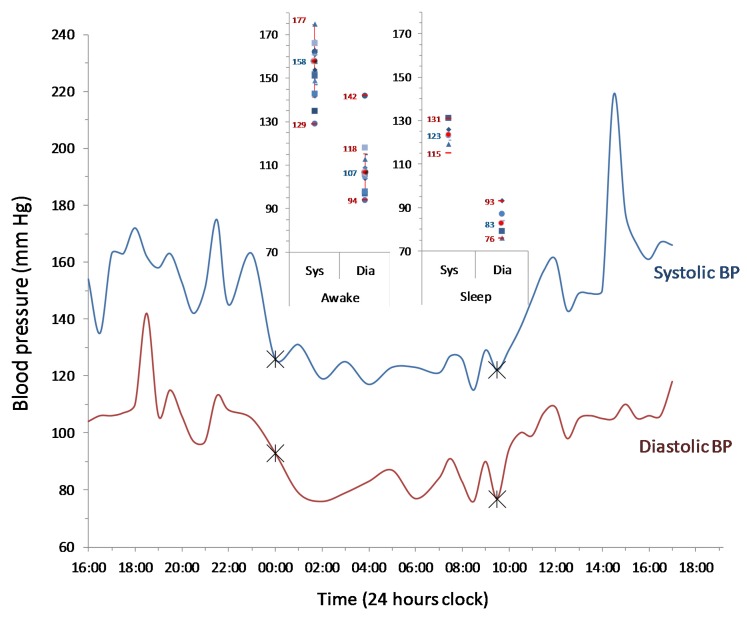
Twenty-four hour ambulatory blood pressure measurement in the subject. The lines represents systolic/diastolic blood pressure recorded over a 24 h period, where the measurements during sleep shows a nighttime dipping (indicated by crossed-marks). The inset shows a distribution of measurements (filled symbols) with minimum/maximum (red bars) and average readings (red filled-circles) in the same subject during sleep/awake cycles, where “Sys” and “Dia” refer to systolic and diastolic blood pressures, respectively.

Here, we present a 35 year old male (Table 1) living in North England with a South Asian background (Pakistan), with a family history of diabetes and hypertension, and a history of recurring renal stones, recently diagnosed with high blood pressure on a 24 h ambulatory blood pressure (BP) monitoring (awake averages: 158/107 and during sleep: 123/83). The patient is otherwise healthy, is not on any medication but takes occasional over-the-counter antihistamines for seasonal hay fever, and has never smoked or consumed alcohol. This subject is a typical example of a “healthy”, young South-Asian subject living in England. The subject has normal fasting glucose and HbA_1C_. The two-hour post-oral glucose load (75 g) serum glucose level was also normal, but his serum insulin level was significantly raised (354.4 pmol/L), suggesting a remarkable insulin resistance. This surge in serum insulin also corresponded to the postprandial rise in blood pressure in this subject studied at another occasion (11:00 am, Figure 1). The patient was found with elevated liver enzymes during screening and, on further investigation, liver was found patchy in echo texture on ultrasound, suggesting a non-alcoholic fatty liver disease (NAFLD). Other investigations including electrolytes, C-reactive protein, fasting blood sugar, glycated haemoglobin, full blood count, thyroid-stimulating hormone, autoantibodies, bone profile and urine protein, urine metadrenaline, and serum lipid profiles were unremarkable.

Following the reports of NAFLD and post-glucose load hyperinsulinemia, the subject undertook a low-carb diet program [4,5,6,7] that included no added sugar on foods and drinks, minimum or occasional intake of starchy carbohydrates, having grilled food instead of fried, changing to wholemeal, increasing overall fibre intake, avoiding animal/saturated fats and having polyunsaturated fats, taking more fish and egg into diet, and grazing nuts (mainly cashews, walnuts, pecans, and almonds) during work hours to curb the urge to eat. The subject achieved a significant reduction in waist circumference within weeks, possibly a good indication of losing abdominal fat. On annual review, his liver enzymes and post-glucose load insulin levels were back to normal, and waist circumference, body weight, and BMI were significantly reduced (Table 1), suggesting that the insulin resistance and fatty liver were reversed. The subject lost over 13% of his weight (although not intended, as the patient was not overweight), but this was not surprising and have already reported in various studies [4,5,6,7]. There was, however, no significant change in blood pressure in this subject albeit prior reports of reduction in BP with weight loss in non-obese Asians, such as Japanese [8]. Although baseline lipid profile was normal, there was a further reduction observed in triglycerides, non-HDL cholesterol, cholesterol/HDL ratio, and an increase in serum HDL on annual review (Table 1). 

## 3. Discussion

It is evidenced from the literature that many South Asians, even at a young age, with hypertension and a family history of diabetes have a significantly high risk of developing diabetes [9]. It has been reported that a significant population with relatively lower BMI and waist circumference, similar to this subject, have a substantial risk of developing diabetes [1]. In addition to have a lower BMI, it is not very common to have a substantially high waist circumference (the usual cut-off of >35 inch or 90 cm). In a prospective epidemiological study, 75% of non-obese Bengali Indians with NAFLD were found to have a BMI <25 kg/m^2^ [10]. It is, therefore, expected that many South Asians having undiagnosed early stages of NAFLD have a potentially higher risk of developing diabetes or progressing towards NASH are unaware of the condition, so a major lifestyle intervention will not be encouraged, which may otherwise prove useful for this population at this stage. 

Zelber-Sagi et al. have reviewed the effects of various dietary interventions on hepatic fat, including various types of fats and the association between added sugar in everyday foods, fast-food-rich Western dietary patterns, and NAFLD [11]. Unwin have also criticised the current guidelines of encouraging over-utilisation of complex carbohydrates, such as starchy foods, which may potentially have a higher glycaemic index than the table sugar [4]. In his low-carb trial on 19 type 2 or pre-diabetes patients, a 9% reduction in body weight was found with an average of 15 cm reduction in waist circumference. His patients also benefited with an improvement in their blood pressures (systolic 148 ± 17 to 133 ± 15 mmHg, *p* < 0.005; diastolic 91 ± 8 to 83 ± 11 mmHg, *p* < 0.05) and a reduction in serum gamma-GT (75.2 ± 54.7 to 40.6 ± 29.2 U/L) and serum cholesterol (5.5 ± 1.0 to 4.7 ± 1.2 mmol/L, *p* < 0.01). A 9% weight loss was also reported in a recent randomised controlled trial on 227 overweight or obese adults with type 2 diabetes undertaking a low-carbohydrate diet compared to only 2.5% with control; the glycaemic control was also improved in subjects on a low-carb diet [5]. In another randomised weight intervention trial, Mayer et al. reported that weight loss among subjects undertaking either a low-carb or a low-fat diet was similar, but those on a low-carb diet also benefited from an improved glycaemic control and a significant antiglycemic drug dose reduction [7]. Similar reports of “deprescribing” antidiabetic medications have been reported in patients with type 2 diabetes undertaking intensive lifestyle change programs [12]. 

It has been reported that diets rich in fruits, vegetables, whole grains, dairy, and unsaturated fats are associated with a lower prevalence of metabolic syndrome. Romero-Gómez in a recent review have also suggested that lifestyle intervention and dietary modulations are useful across the wide spectrum of NAFLD. The Mediterranean diet, characterized by low carbohydrates (sugars or refined carbohydrates), and rich in monounsaturated/omega-3 fatty acids and antioxidants (polyphenols), has been found useful in reducing liver fat to prevent the disease progression [6,13]. Giugliano and Esposito have suggested that Mediterranean diet could also be beneficial in chronic inflammation associated with visceral obesity, type 2 diabetes, and metabolic syndrome [14]. Recently, the Mediterranean diet was also found superior to a low-fat diet in reducing intrapericardial-fat burden in a randomized controlled trial [15], hinting their potential in preventing cardiovascular events.

## 4. Conclusions

NAFLD is common in non-obese South Asians with considerably lower BMI and waist circumference without obvious manifestations of abdominal adiposity. Dietary interventions have already proven beneficial in overweight or obese subjects with fatty liver. This study encourages that dietary interventions can also be beneficial to individuals with fatty liver who are not yet overweight. This, however, warrants large cohort studies to substantiate these findings.

## Figures and Tables

**Table 1:** Subject characteristics at baseline and after lifestyle interventions.

Description	Baseline	Annual Review
Age (year)	35	36
Body weight (Kg)	59	51
Height (cm)	163	~
BMI (Kg/m^3^)	22.3	19.1
Waist circumference (inches)	34	29
Fasting glucose (mmol/L)	4.6	–
HbA_1c_ (mmol/mol)	37	33
Fasting glucose (2-h post glucose load), (mmol/L)	4.0	4.3
Insulin (2-h post glucose load), (pmol/L)	354.4	171.8
Total bilirubin (umol/L)	11	12
Alkaline phosphatase (iU/L)	130	97
ALT (iU/L)	75	17
Lipid profile		
Serum cholesterol (mmol/L)	5.7	5.7
Serum triglycerides (mmol/L)	1.5	1.0
Serum HDL (mmol/L)	1.3	1.6
Serum cholesterol/HDL ratio	4.4	3.6
Serum LDL (mmol/L)	3.7	3.6
Non-HDL cholesterol (mmol/L)	4.4	4.1

Note: ~ unchanged; – not tested.
